# Advanced Approach of Multiagent Based Buoy Communication

**DOI:** 10.1155/2015/569841

**Published:** 2015-08-04

**Authors:** Gediminas Gricius, Darius Drungilas, Arunas Andziulis, Dale Dzemydiene, Miroslav Voznak, Mindaugas Kurmis, Sergej Jakovlev

**Affiliations:** ^1^Institute of Mathematics and Informatics, Vilnius University, Akademijos Street 4, 08663 Vilnius, Lithuania; ^2^Department of Informatics Engineering, Faculty of Marine Engineering, Klaipeda University, Bijunu Street 17-206, 91225 Klaipeda, Lithuania; ^3^Institute of Digital Technologies, Faculty of Social Technologies, Mykolas Romeris University, Ateities Street 20, 08303 Vilnius, Lithuania; ^4^Department of Telecommunications, Faculty of Electrical Engineering and Computer Science, VSB-Technical University of Ostrava, 17. Listopadu 15, 708 00 Ostrava, Czech Republic

## Abstract

Usually, a hydrometeorological information system is faced with great data flows, but the data levels are often excessive, depending on the observed region of the water. The paper presents advanced buoy communication technologies based on multiagent interaction and data exchange between several monitoring system nodes. The proposed management of buoy communication is based on a clustering algorithm, which enables the performance of the hydrometeorological information system to be enhanced. The experiment is based on the design and analysis of the inexpensive but reliable Baltic Sea autonomous monitoring network (buoys), which would be able to continuously monitor and collect temperature, waviness, and other required data. The proposed approach of multiagent based buoy communication enables all the data from the costal-based station to be monitored with limited transition speed by setting different tasks for the agent-based buoy system according to the clustering information.

## 1. Introduction

There are a variety of tools to monitor and evaluate Baltic Sea hydrometeorological data, but most received information has low spatial coverage and low level of detail in time [1]. Sea wave height, water temperature, and underwater noise data, used for many practical applications, are usually obtained from three sources: buoy measurements, model calculations, and ship observations. Compared to other data acquisition methods, buoy measurements are the most reliable and readily data source available continuously for years [2]. Basically, the network of buoys is involved in mapping the temperature, wave height, and underwater noise at a buoy location using the data retrieved from other buoy locations [3]. However, many hydrometeorological data measurements using sea buoys can be lost due to malfunctions, maintenance, connection problems, or dubious data recorded by the buoy. In order to ensure greater reliability of data collection, it is necessary to develop a distributed information system, predicting complex situations and supporting decision-making processes. Information provided from such system is important for decision-makers and is needed to ensure the provision of information for decision-making institutions [4–6]. An important feature of the buoy network is the ability to monitor, collect, and evaluate wide spatial coverage and real-time hydrometeorological data of the Baltic Sea [5]. A hydrometeorological information system is faced with great data flows, but the data levels are often excessive, depending on the observed region of the water. Therefore, current traditional methods are no longer sufficient to ensure the rapid collection of data and valuable information extraction.

The purpose of this study is to show the possibilities of developing a hydrometeorological data collection system (HMDCS) involving advanced technologies such as multiagent based interaction and data collection between several monitoring system nodes (i.e., buoys) based on self-organizing maps (SOM). The experiment is based on the design of the inexpensive but reliable Baltic Sea autonomous monitoring network (buoys), which would be able to continuously monitor and collect temperature, waviness, and other required data. Moreover, it has the ability to monitor all the data from the coast-based station with limited transition speed by setting different tasks for the agent-based buoy system according to the SOM.

## 2. Sea Hydrometeorological Data Monitoring

Nowadays, there are numerous and varied designs for autonomous systems used for meteorological and oceanographic monitoring with different integration degrees. The buoy network system used in the Canary Islands is one of them [7]. It has a control center that manages the transmission communications and provides data in a useful form to diverse socioeconomically important sectors which make exhaustive use of the littoral, and data from the buoys are used to manage the coastal environment. The buoys monitor water temperature, salinity, dissolved oxygen, hydrocarbons, and other characteristics, which they can measure when equipped with other sensors such as a fluorometer and a turbidimeter, and each buoy is also able to communicate via GSM modem. Following a programmed sampling rate (every hour), the ECU sends to the central receiver unit a SMS message, which includes a sensor data set, GPS position, and battery level. However, deeper analysis of the data has shown that such a sampling rate is not sufficient, which means that the data transmit protocol must be reevaluated.

In order to provide greater hydrometeorological data monitoring reliability and faster data retrieval, a variety of sensory systems networks [8–10] have been proposed, such as communication technologies that enable communication between sensor nodes [10], systems for communication between maritime platforms like vessels, commercial ships, or buoys [9], and real-time monitoring of the underwater environment where an acoustic mesh network is located between the underwater sensor networks and the central monitoring system [8]. The proposed models can solve various problems but require more flexible solutions for complex data transfer problems. This problem can be solved by developing an active autonomous sensor multiagent based system, which is able to combine data processing methods according to the situation.

## 3. Hydrometeorological Data Sensory System

### 3.1. Temperature Data Collection

During the investigation stage of the HMDCS development, several types of temperature sensors were compared. The comparison possibilities are made by analyzing their parameters according to the technical specifications presented in datasheets.

After a comparative analysis of the temperature sensors, we selected the DS18B20 digital sensor. This digital temperature sensor can measure temperatures within the range from −55°C to +125°C at 12-bit precision, with accuracy −0.50°C [11]. However, after additional calculations, it is possible to reduce the temperature measurement error down to 0.10°C. The most attractive feature is the fact that these sensors have already been calibrated at the factory and their accuracy error is ±0.5°C in the range from −10°C to +85°C and ±2°C error over the operating range (55°C to +125°C). Sensor supply voltage is in the range of +3 to +5.5 V. In standby mode, current consumption is close to zero (less than 1 *μ*A), while temperature conversion power use is about 1 mA. The measurement process lasts no more than 0.75 sec. The DS18B20 communicates over a 1-Wire bus that by definition requires only one data line (and ground) for communication with a central microprocessor. In addition, the DS18B20 can derive power directly from the data line (“parasite power”), eliminating the need for an external power supply. Each DS18B20 has a unique 64-bit serial code, which allows multiple DS18B20s to function on the same 1-Wire bus. Thus, it is simple to use one microprocessor to control many DS18B20s distributed over an area of few square meters (in our case they are used to measure temperature in different depth of the sea). This part has already become the corner stone of many data logging and temperature control projects.

### 3.2. Waviness Measurements

At present, sea and ocean waviness measurements use a variety of methods, depending on the geographic region, measuring accuracy, and general tasks [12]. The main and most commonly used are as follows:

- ultrasound-based sensors:
  1. pros: suitable for measuring waves with a height of over 5 meters,
  2. cons: significant measurement errors,
- rheostat-type structures:
  1. pros: allow you to get fairly accurate data,
  2. cons: because of their design features they have a short lifetime,
- satellite image analysis:
  1. cons: due to the inherent large errors, this can be used only for ocean waviness measurements,
- GPS system:
  1. cons: not suitable for measuring waves with a height of 0.5–2.0 meters,
- accelerometer and gyroscope design:
  1. pros: small measurement errors, easy implementation.

For our experiment the couple of accelerometer and gyroscope was used. Based on the experience of other scientists [1], accelerometer data were processed by removing the component of gravity, according to the following:

(1) $$\begin{array}{c} \left[\begin{array}{c} X_{E}\\ Y_{E}\\ Z_{E} \end{array}\right]=\left[\begin{array}{ccc} a_{1} & b_{1} & c_{1}\\ a_{2} & b_{2} & c_{2}\\ a_{3} & b_{3} & c_{3} \end{array}\right]\left[\begin{array}{c} X_{S}\\ Y_{S}\\ Z_{S} \end{array}\right]. \end{array}$$

Here, *X*_*S*_, *Y*_*S*_, *Z*_*S*_ represent the accelerations measured in the sensor frame, *X*_*E*_, *Y*_*E*_, *Z*_*E*_ are the accelerations rotated into the earth coordinate frame, and the direction cosines for the above transformation are in terms of the Euler attitude angles.

The coefficients *a*, *b*, and *c* are calculated using the following formulas:

(2) a1=cos⁡θcos⁡ψ,b1=sinφsinθcos⁡ψ−cos⁡φsinψ,c1=sinφsinθcos⁡ψ+cos⁡φsinψ,a2=cos⁡θsinψ,b2=sinφsinθcos⁡ψ+cos⁡φsinψ,c2=sinφsinθcos⁡ψ−cos⁡φsinψ,a3=−sinθ,b3=sinφcos⁡θ,c3=cos⁡φcos⁡θ.

Here, *θ*, *ψ* and *φ* are data from the gyroscope. After the accelerations have been rotated into the earth frame, the earth-referenced accelerations of the buoy are given by

(3) AX=−gXE,AY=−gYE,AZ=g1−ZE,

where *A*_*X*_, *A*_*Y*_, and *A*_*Z*_ are accelerations with eliminated gravity force along the earth-oriented *X*-, *Y*-, and *Z*-axes.

### 3.3. Data Transmissions

After comparing the most popular data transmission protocols such as Bluetooth, UWB, ZigBee, Wi-Fi, and others [5], it was decided that ZigBee is the most suitable transmission protocol for such a task (low-cost, low power, mesh network support). So, this mesh-type network protocol was used for developing the HMDCS buoy network. ZigBee is an open standard for short-range wireless networks based on the Physical Layer and the Media Access Control from IEEE 802.15.4, focusing on minimizing the overall power consumption and at the same time maximizing network reliability [13].

The ZigBee protocol offers three kinds of devices to form a PAN (personal area network):

1. end-devices, which periodically collect data and transmit it,
2. routers: they collect data from end-devices and forward it to the destination (like another router or to the final coordinator),
3. coordinator: one of the routers in a PAN is usually configured as a coordinator. The main function of the coordinator is the parameterization and management of the PAN, and the collection of network data.

**Figure 1:**
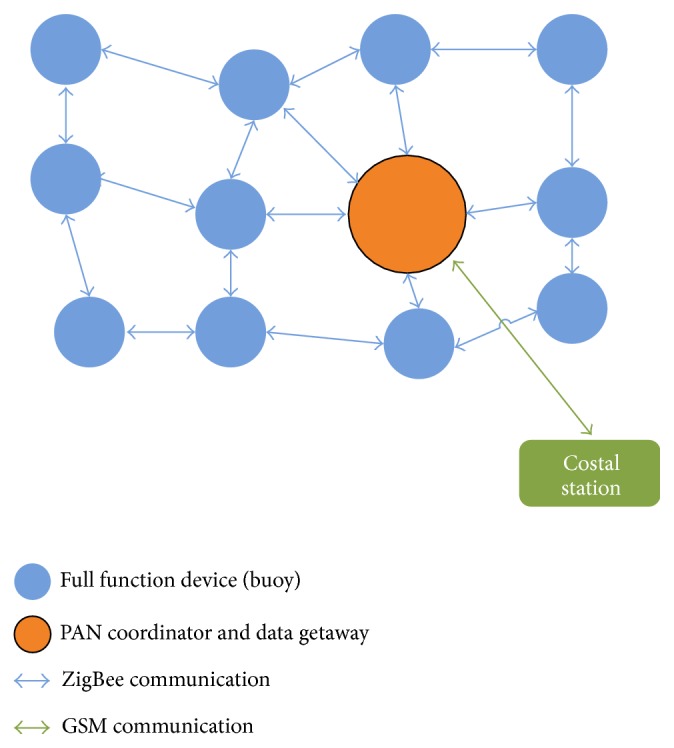
Mesh network.

In our case, we used so-called “full function devices” which collect data and work as a router and coordinator, which manages the PAN network and sends collected data via GSM to a coastal station (Figure 1). The following ZigBee network configuration was used for transmitting data to the coastal station.

### 3.4. Buoy Power Management

Power requirements for the electronic buoy system are 5 V DC at 76 mA in active cycle (active sensors and microcontroller are calculating data; transmitter is sending information) and about 27 mA at passive cycle (microcontroller is in sleep mode, and only the receiver is powered up for wake up using external interrupt). Active cycle lengths is about 5 seconds in every 10 minutes, so the duty cycle of the buoy is 0,0083. The buoy power supply consists of a battery bank of 18 AA type Ni-MH battery cells, arranged in 3 parallel groups of 6 cells connected in series. The capacity of each battery used in our buoy system is 2200 mAh so the capacity of each group of 3 batteries combined in parallel groups is 6600 mAh at 7.2 V. Expected lifetime of such configuration system at 25°C temperature would be about 10 days. Batteries placed in the bottom of the buoy also serve as ballast.

## 4. Agent Action Distribution Using SOM

**Figure 2:**
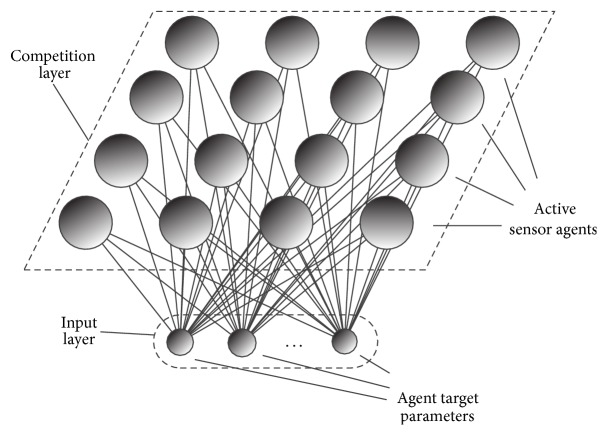
SOM for autonomous agents-based sensory system.

The proposed multiagent sensory system is based on the goal of task distribution for agents according to action similarities. This can be implemented applying self-organising map neural networks (SOM). A SOM defines a two-dimensional nonlinear manifold as a regular array of discrete points. In this way, the application of unsupervised learning allows a multidimensional vector represented in two-dimensional output space. The SOM output layer neurons retain a topological structure according to internal data structure. A typical SOM neural network architecture is shown in Figure 2. The input nodes represent the parameter vector, which according to the similarity is projected in the two-dimensional output space—the competitive layer. The input layer represents the parameters of the agents' target selection, and the competitive layer represents the autonomous agents-based sensory system.

In order to get the topological structure of the SOM, a training process should be applied. Each unit in the competition layer array is associated with a parametric reference vector weight of dimension *n*. Each input vector is compared with the reference vector weight *w*_*j*_ of each unit. The best match with the smallest Euclidean distance is defined as the response, and the input is mapped onto this location. Initially, all reference vector weights are assigned to small random values and are updated as [14]

(4) $$\begin{array}{c} \Delta w_{j}=\alpha_{n}\left(t\right)h_{j}\left(g,t\right)\left(x_{i}-w_{j}\left(t\right)\right), \end{array}$$

where *α*(*t*) is the learning rate at time *t* and *h*_*j*_(*g*, *t*) is the neighborhood function from winner unit neuron *g* to neuron *j* at time *t*. In general, the neighborhood function decreases monotonically as a function of the distance from neuron *g* to neuron *n*. This decreasing property is a necessary condition for convergence [14].

SOM competition layer nodes correspond to individual agents as active sensory nodes, which are able to process data at a different level (filtering, sampling, transfer, and other). The capacity of the wireless network, the data capture excess in the central database, and so on depend on these characteristics. Assuming that each agent as an active buoy sensor node performs different actions, the central unit can distribute tasks for the agents in accordance with their capabilities and the required information. In this case, we use three parameters as the inputs for the SOM, which determine the actions performed by the agents—the significance of the measurement data, hydrometeorological characteristics of interest, and the number of the sampling rate. These parameters as appropriate capability are predefined for every active sensor agent in the SOM's competition layer. Under these settings, the actions are distributed for the agents according to the common goal and the capability of each agent. For example, if we need raw data, the task will be forwarded to agents that have a high data transfer bandwidth but do not have filtering capabilities.

## 5. Multiagent System Model for Hydrometeorological Sensory System

**Figure 3:**
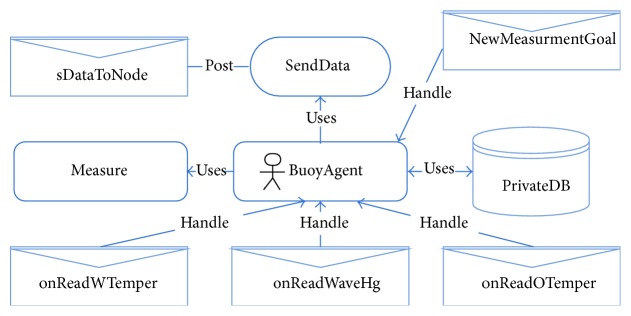
Buoy agent schematic.

**Figure 4:**
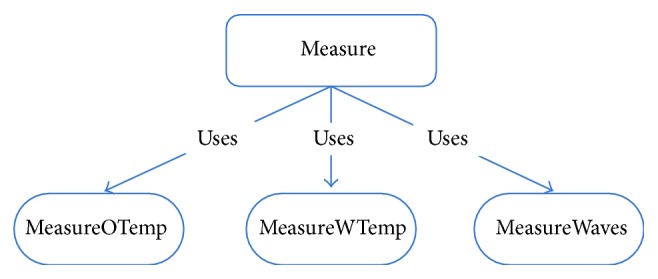
Measure data capability.

For proper buoy operation, a multiagent type system was designed. The agent software was developed using a multiagent framework and works internally in the buoy.  Figure 3 shows one buoy agent example. The buoy agent has one main goal: measure data and different tasks given by posting a newMeasurmentGoal message from coordinator (SOM network). The buoy agent can read new data using capability Measure (Figure 4). Once the sensor has read the data, the messages onReadWTemper (for water temperature), oReadOTemper (for weather temperature), and onReadWaveHg (for wave height) occur.

The buoy agent stores data in the local DB and if necessary, it is able to post it to other agents via the ZigBee network using the plan SendData.

## 6. Results and Discussion

For sea wave height, five different measurement methods were analyzed: using an ultrasonic sensor, a rheostat-type sensor, accelerometer and gyroscope sensors, satellite photos, and GPS data [12]. For data transmission from buoys to the main station different transmission methods and protocols were analyzed, but most focused on mesh-type wireless networks and agent-based communication methods [11, 15].

**Figure 5:**
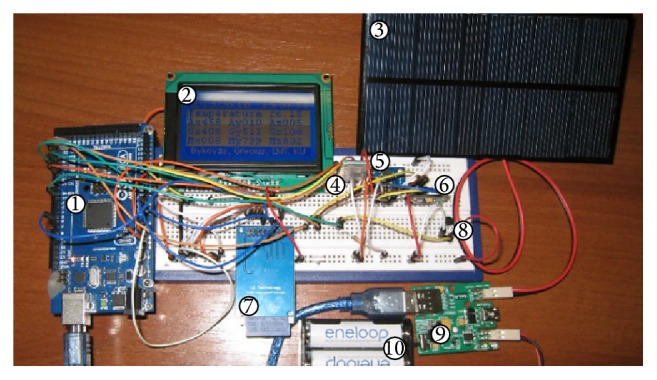
Buoy electronic system prototype.

**Figure 6:**
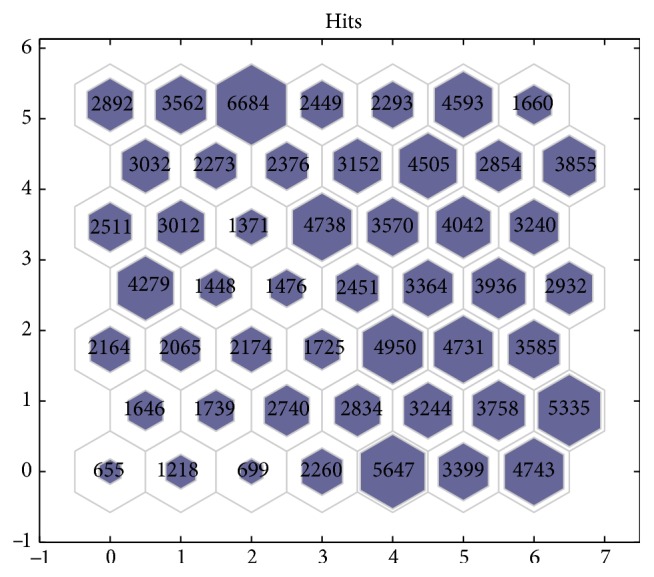
The distributed sensory node priorities using the SOM neural network.

For testing purposes, an experimental buoy sensory system was developed. The core component of the prototype is the Arduino Mega platform with an ATmega2560 microcontroller which operates at 16 MHz clock frequency (Figure 5-(1)). The experimental buoy system is powered by solar power supply, which also recharges the Ni-MH batteries, which allow the buoy sensory system to operate at night (Figure 5-(3), (9), and (10)). Buoy status is shown on an LCD display (Figure 5-(2)). XBee Pro modules (Figure 5-(4)) implement communication via the ZigBee protocol and have 10 mW transmission power and according to the specifications, an expected distance of about 1–1.5 km outdoors. Temperature measurements (underwater and weather) are implemented using a DS18B20 sensors array connected to a 1-wire network (Figure 5-(5)). Data logging is to MMC (Figure 5-(7)). The wave height is measured using an MPU6050 (Figure 5-(6)) accelerometer/gyroscope and calculated by the provided method.

The constructed prototype was placed in a hermetic housing and tested offshore in the Baltic Sea. The construction design and electronics solutions look very promising: one buoy's electronics cost only about 100 EUR, and the experimentally tested point to point network with 10 mW Xbee modules in open sea has a transmission distance of at least 900 m (it is enough to build a mesh-type buoy network).

According to the Baltic Sea Monitoring Data Base [16] a hydrometeorological mesh-type data collection network was established, which enables the performance evaluation of each sensor node. This evaluation allows the coastal central station to distribute the agent performance according to the amount of required data.  Figure 6 shows the distribution of sensory node priorities using the SOM neural network.

Each sensor node priority defines the importance of the measurements and the amount of data transmitted; that is, a sensor node with higher priority requires the agent to transmit larger amounts of data, which should allow a more accurate assessment of the sea region of interest.

## 7. Conclusions

This paper presents the possibilities of developing a hydrometeorological data collection system (HMDCS) involving advanced technologies such as multiagent based interaction and data collection between several monitoring system nodes (i.e., buoys) based on self-organizing maps (SOM). The proposed solutions of HMDCS look very promising because of the inexpensive but reliable Baltic Sea autonomous monitoring network (buoys), which is able to continuously monitor and collect temperature, waviness, and other required data. The multiagent type system was designed to monitor data from a coastal-based station with limited transition speed by setting different tasks for the agent-based buoy system according to the SOM.
